# Woven Coronary Artery Anomaly: An Incidental Finding and Literature Review

**DOI:** 10.1155/2022/3235663

**Published:** 2022-04-14

**Authors:** Bdoor Bamousa, Taher Sbitli, Tahir Mohamed, Khalid Al Johani, Ali Almasood

**Affiliations:** ^1^Department of Medicine, Alfaisal University, Riyadh, Saudi Arabia; ^2^Heart Center, King Faisal Specialist Hospital & Research Center, Riyadh, Saudi Arabia; ^3^Cardiac Science Department, King Saud University, Riyadh, Saudi Arabia

## Abstract

Woven coronary artery anomaly is a rare description of an epicardial vessel segment that divides into multiple intertwining segments with eventual convergence of the distal vessel. We present our case, a 57-year-old male with an incidental woven coronary artery anomaly found during work-up investigations for a possible lung transplant, and we conduct a literature review on woven anomaly cases reported from 1988 to 2021 and provide a thorough analysis of its diversified clinical presentation. Imaging identification and various treatment modalities are also discussed.

## 1. Introduction

Woven coronary artery anomaly is a rare and sporadic disorder that has been previously described as a benign pathology. Recently, there has been an increase of reported “malignant” or ischemic forms of this disorder in the literature. This anomaly entails the division of the epicardial coronary artery into multiple channel segments that converge distally. It has been associated with the formation of a thrombus, consequently giving rise to more serious clinical sequelae. Herein, we report a case of a 57-year-old male patient with a woven anomaly of the coronary artery that was treated with percutaneous coronary angioplasty.

## 2. Case Report

**Figure 1:**
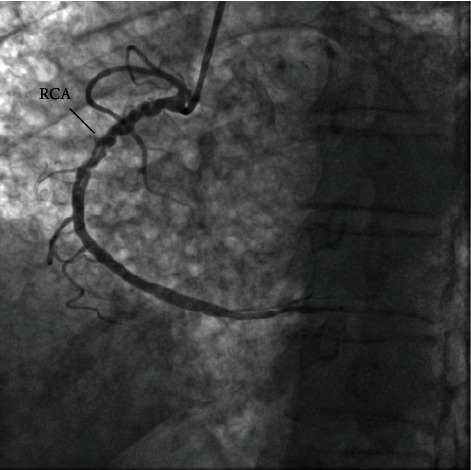
Left anterior oblique (LAO) view of right coronary artery (RCA), demonstrating braid-like lesion.

**Figure 2:**
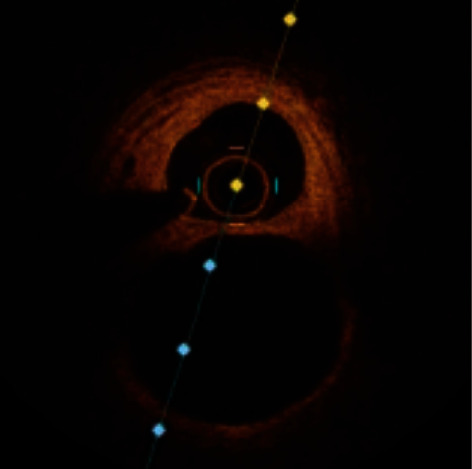
Optical coherence tomography (OCT) of right coronary artery (RCA) showing multiple channels.

**Figure 3:**
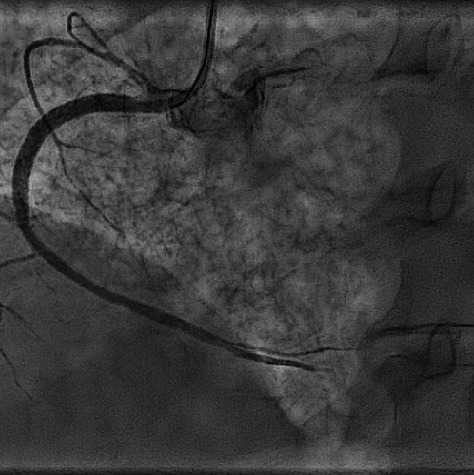
Right coronary artery (RCA) postpercutaneous intervention (PCI).

A 57-year-old male, with a known case of idiopathic pulmonary fibrosis, diagnosed 7 years ago, presented to our emergency room due to shortness of breath and productive cough for the last 2 months. He is a previous smoker for 25 years and has quit 2 years ago. He has no history of hypertension, diabetes, or any other coronary artery disease risk factors. During his admission, he underwent work-up for possible lung transplantation. His blood pressure was 114/71 mmHg. An electrocardiogram showed normal sinus rhythm. Echocardiography demonstrated findings of moderately severe pulmonary hypertension, mild dilatation of the right atrium, and normal left ventricular systolic function without any regional wall motion abnormalities, with an ejection fraction of >55%. He underwent a coronary angiogram as a routine procedure prelung transplantation which demonstrated branching of the proximal segment of the right coronary artery (RCA) into thin channels that converge distally into a normal conduit (Figure 1). Optical coherence tomography (OCT) was performed and documented the finding of a braid-like woven coronary with multiple channels within the vessel (Figure 2). Left anterior descending artery (LAD) and left circumflex coronary artery (LCX) vessels had mild ectasia, with otherwise normal Thrombolysis In Myocardial infarction (TIMI) III flow. According to the hospital protocol and for the patient to remain on the lung transplantation listing, coronary intervention had to be done; therefore, percutaneous coronary intervention (PCI) was performed; an Asahi Sion blue wire was used to cross the woven lesion successfully, the lesion was predilated, and a DES stent was placed and deployed successfully (Figure 3). Post PCI, the patient was put on dual antiplatelet therapy. He was reevaluated in 3 months, during which he was admitted for his lung transplant.

## 3. Discussion

Woven coronary artery anomaly is defined as a condition in which an epicardial vessel is divided into multiple thin vessel channels that share the same tunica proximally and converge into one lumen without any disruption of blood flow distally [1]. Beyond the intertwining or “woven” segment which averages at about 2.2 cm with a range of 1-5 cm, blood flow is TIMI-III in the involved vessel [2]. A limited number of cases have been published on this anomaly. The recent rise in WCAA reports may be due to the greater usage and advancement of intravascular imaging modalities that have made the anomaly easier to detect.

The first ever reported case of WCAA was in 1988. Sane and Vidaillet published a case report describing a 55-year-old female with rheumatic heart disease and valvular disorders. She presented with what were presumably symptoms of congestive heart failure with involvement of the mitral valve, but upon further evaluation, the proximal RCA was visualized and said to have a “figure 8” pattern on the arteriogram [3].

We have conducted a literature review that encompasses all published cases of WCAA from 1998 until June 2021, with a total of 37 cases. The demographic's information and the clinical details of these cases are in Tables 1 and 2. The mean age was found to be 53.2 ± 12.4 years, with the male gender being predominantly affected; out of the 37 patients, only 2 were female (male to female ratio of 17.5 : 1). All listed patients are adults, except for one case, a 9-month-old infant with Kawasaki disease [4].

The most common vessels involved in WCAA are RCA (70.2%), LAD (32.4%), and LCX (18.9%). WCAA malformation can affect more than one vessel at once and is more frequently found within a segmental artery [5]. The percentage of 2 or more vessels involved concurrently is 17.9%.

Multiple theories have been proposed regarding the etiology of WCAA, despite the exact cause being unknown. In the literature, there have been 2 main proposed theories regarding pathology. Some believe that this disorder is congenital and more specifically sporadic as no reported cases are identifying a genetic predisposition or hereditary cause [1] [6]. Others believe that it is a consequence of spontaneous cardiac dissection or recanalized thrombus [1, 7]. The causes of WCAA can be vasculitic disorders such as spontaneous coronary artery dissection, recanalized thrombus, or Kawasaki disease, and it may be associated with long-term chronic conditions such as rheumatoid arthritis [1] [2] [3] [6, 8]. It is important to understand that WCAA can be due to spontaneous cardiac dissection or recanalized thrombus but can also mimic those pathological states. It is therefore advised to keep these similar pathologies in your list of differential diagnoses when considering a potential case of a woven coronary artery anomaly [9]. Despite an unknown pathophysiology, it has been postulated that certain growth factors involved in arteriogenesis and angiogenesis are key in the underlying pathophysiology of this disorder [1, 6]. Overall, the most commonly supported theory in the literature is that WCAA is of congenital origin [6, 10].

We believe that WCAA can be both congenital and acquired, provided that the typical characteristics of multiple thin vessels share the same tunica proximally and converge into one lumen distally without any disruption of blood flow. The presence of 3 distinct vessel wall layers indicates a congenital origin, while the preexistence of cardiac dissection or recanalized thrombus may hint at an acquired state of the anomaly.

Patients with the underlying woven anomaly of the coronary arteries tend to be asymptomatic for years and may even go undetected. This led to the anomaly being deemed as benign in the literature. However, there is now a rise in cases reported of patients presenting with acute coronary syndrome, ischemic stroke, myocardial infarction, or sudden cardiac death [7] [10–12]. Underlying risk factors may contribute to a malignant presentation of WCAA. The prevalence of predetermined risk factors in the patient population of woven coronary artery anomalies in the literature is as follows: 21.6% of patients have hypertension, 13.5% have dyslipidemia, 27% are smokers, and 16.2% have a preexisting history of ischemic heart disease.

The literature proposes WCAA as the culprit responsible for thrombus formation [11, 13]. The intertwining of the thin channels gives rise to an environment prone to thrombus formation [4]. A case report in 2018 strongly suggests that WCAA should be categorized under the umbrella of atherosclerotic disease, as complications of this disorder require similar treatment [7]. Up until 2012, reported cases of WCAA have not been associated with ischemia. The first reported case of WCAA causing ischemia was a 48-year-old man who presented with myocardial infarction due to the woven anomaly. This patient had a previous diagnosis of spontaneous coronary artery dissection 6 years before the MI, which may support the hypothesis that WCAA can be caused by coronary dissection [11]. Spontaneous dissection of the artery initially forms pseudolumens. It is theorized that over time, these pseudolumens join and form true lumens which spiral around each other in a woven formation, predisposing the patient to a thrombus [10, 11] Closer inspection and follow-up is required for such patients, to prevent adverse or life-threatening complications.

Coronary angiography is deemed as the gold standard method of identifying and diagnosing WCAA [2, 14]. Without familiarization of the disorder with imaging, woven anomaly may be missed [5]. WCAA can be viewed as a filling defect and as previously mentioned can appear similar to spontaneous coronary dissection or recanalized thrombus, with the images being described as “honeycomb,” “spiral” [8], “braid-like,” or having a “figure 8” pattern [3, 8].

A published case series in 2020 argues that some of the case reports on woven anomalies are misdiagnosed and could be recanalized organized thrombi. It further states that merely doing an angiogram is not sufficient to determine the diagnosis as both these pathological states will present as a “braid-like lesion” [15].

Optical coherence tomography (OCT) and intravascular ultrasound (IVUS) [16] are intravascular imaging modalities that will provide a definitive diagnosis with a high-resolution illustration of the lumen of the vessel and the three layers of the vessel wall [5, 17].

Evidence supporting the theory that WCAA is of congenital origin can be supported by using OCT. In a woven anomaly, OCT findings will demonstrate intertwined thin segments separated by fibrous tissue with no cross-communication between those segments, undisrupted arterial wall integrity without dissection, and high signal intensity and low signal attenuation [5, 18, 19]. You may also visualize a thrombus formation in more malignant cases of woven anomaly [19]. Cross-communication is a key feature in distinguishing WCAA and recanalized thrombus. On OCT, the recanalized thrombus has been described as a “lotus-root” or “swiss-cheese” appearance due to the presence of multiple interconnected channels within the thrombus [20, 21]. This is unlike a congenital woven anomaly, which has no cross-communication between channels [22].

Because of the interchangeable usage of description terms, a key feature distinguishing these pathologies will help prevent any misdiagnosis. We believe OCT to be an essential tool for confirmation and avoidance of any undesirable operative complications due to misdiagnosis. OCT may not be suitable for all cases. In some instances, OCT wires may be difficult to penetrate through the complex structure of a woven anomaly associated with a thrombus or may simply be unavailable at the given facility. One case was able to overcome this by utilizing a patient's previous angiogram 3 years prior to deduce the location of the thrombus; the previously done angiogram revealed a hazy lesion followed by stenosis in the RCA, which was medically treated. Based on the assumption that the woven anomaly must have been caused by progression and recanalization of that thrombus, a PCI was performed afterward in addition to an IVUS [23].

Treatment modalities of WCAA vary from conservative percutaneous intervention to bypass surgery. Asymptomatic patients should be kept under observation especially if there is no evidence of ischemia. However, once symptoms such as angina are noted, patients must undergo further testing to confirm ischemia before deciding upon interventions such as surgical or percutaneous revascularization [21–23]. A method that can be helpful in deciding upon an intervention is the usage of fractional flow reserve (FFR) [24]. FFR determines the adequacy of coronary blood flow using a formula that takes the distal coronary pressure of a stenosed vessel and divides it by aortic pressure. In 2019, a study applied fluid dynamic principles to understand the variables affecting the pressure drop along a woven coronary artery. The number of channels and length of the diseased segment were found to have an inversely proportional relationship with pressure along the artery. The greater the length and the number of channels, the greater the pressure will drop. This supported their hypothesis that FFR is more effective in determining the adequacy of coronary blood flow as compared to coronary angiography [25].

Before ischemic WCAA was established in the literature, PCI was ruled as an ineffective treatment modality [11]. In our reported case, the decision was made to perform PCI on the RCA, based on clinical judgment and angiographic evaluation of TIMI flow I-II. Adequate blood supply was established in the RCA territory, with a TIMI-III grade flow.

## 4. Conclusion

WCAA is a rare braid-like pathology that can lead to a serious clinical sequela. We believe that it can arise in both an acquired and congenital form. Familiarization and proper identification of this anomaly are necessary with the use of coronary angiogram and more specifically OCT; visualization of the vessel wall layers and lumen will help distinguish a woven anomaly from similar pathologies such as spontaneous coronary artery dissection, recanalized thrombus, or bridging collaterals. Based on the ischemic assessment of the patient, treatment modalities can be chosen. Patients found to have an incidental benign woven anomaly must also be observed, as the anomaly can convert to a malignant variant and cause devastating medical consequences such as acute coronary syndrome or sudden cardiac death.

## Figures and Tables

**Table 1:** A compiled list of case reports published in the literature and demographic's information.

#	Year	Author	# of cases	Age (year)	M/F	Risk factors				Past medical history
						Hypertension	Dyslipidemia	Smoking	IHD	
1	1988 [3]	Sane and Vidaillet	1	55	F	0	0	0	0	RHD, aortic valve replacement, and mitral valve commissurotomy
2	1990 [9]	Berman et al.	1	51	M	0	1	1	0	Family history of premature CAD
3	1995 [26]	Gregorini et al.	3 (3A)	60	NA	0	0	0	0	
			3 (3b)	62	M	0	0	0	0	
			3 (3c)	45	F	0	0	0	0	
4	2000 [27]	Martuscelli et al.	1	42	M	0	0	0	1	Angina and family history of hypercholesterolemia
5	2006 [28]	Kursaklioglu et al.	1	48	M	0	0	0	0	
6	2010 [4]	Yildirim et al.	1	0.75 (9 m)	M	0	0	0	0	
7	2010 [2]	Iyisoy et al.	1	58	M	0	0	1	0	
8	2012 [11]	Soylu et al.	1	48	M		1	0	0	
9	2012 [29]	Tasal et al.	1	60	M	1	0	1	0	
10	2013 [14]	Yuan	1	62	M	1	0	0	1	
11	2013 [30]	Akyuz et al.	1	45	M	1	1	1	0	Carotid artery occlusion
12	2013 [13]	Ayhan et al.	1	42	M	0	0	0	0	
13	2013 [17]	Bozkurt et al.	1	52	M	0	0	0	1	
14	2013 [31]	Oylumlu et al.	1	53	M	0	0	1	0	
15	2013 [19]	Uribarri et al.	1	73	M	0	0	0	0	
16	2013 [32]	Abaci et al.	1	46	M	0	0	0	0	
17	2014 [33]	Acar et al.	1	54	M	0	0	0	0	
18	2015 [34]	Alsancak et al.	1	54	M	0	0	0	0	
19	2015 [35]	Baysal et al.	1	61	M	1	1	0	0	
20	2015 [12]	Chikata et al.	1	75	M	0	1	0	0	Atrial flutter
21	2017 [10]	Val-Bernal et al.	1	39	M	0	0	0	0	
22	2017 [36]	Xing et al.	1	51	M	0	0	0	0	Mitral regurge
23	2018 [7]	Akcay and Soylu	1	41	M	0	0	0	0	
24	2019 [5]	Wang et al.	3(3A)	62	M	1	0	0	0	Atrial fibrillation
			3(3B)	61	M	1	0	0	1	
			3(3C)	66	M	1	0	1	0	
25	2019 [21]	Bi et al.	1	59	M	0	0	0	0	
26	2019 [23]	Wen et al.	1	67	M	0	0	0	0	
27	2020 [1]	Wei et al.	1	67	M	0	0	1	0	
28	2020 [8]	Liu and Li	1	44	M	0	0	0	1	Atrial fibrillation, RHD, and severe mitral regurge
29	2020 [18]	Wang et al.	1	48	M	0	0	0	0	
30	2020 [20]	Uemura et al.	1	53	M	0	0	0	0	
31	2020 [37]	Li et al.	1	47	M	0	0	1	0	
32	2020 [24]	Vilalta et al.	1	59	M	1	0	1	1	
33	2021	Almasood et al.	1	57	M	0	0	1	0	Idiopathic pulmonary fibrosis

Risk factors: 1 = existant risk factor. 0 = non − existent risk factor.

**Table 2:** Outline of the clinical details of the woven anomaly case reports found in the literature.

#	Presenting symptom	ECG	Echocardiography	Ischemia assessment	OCT	IVUS	Woven anomaly vessel	Diseased artery	Treatment	Outcome or F/U

1	Shortness of breath				Not done	Not done	Proximal RCA			
2	Incidental			Exercise ECG: diffuse ST depression in anterolateral and inferior leads+thallium scan: inferoposterior defect	Not done	Not done	Middistal RCA	PDA		
3	ACS			Positive thallium scan	Not done	Not done	LAD, LCX, and OM			
	ACS				Not done	Not done	Proximal LCX	LAD, LCX	PTCA for mid LAD (percutaneous transluminal coronary angioplasty)	
	ACS				Not done	Not done	Distal LAD	LAD		
4	Chest pain				Not done	Not done	Middistal RCA	RCA		
5	Chest pain	Normal	Moderate aortic insufficiency	Stress ECG: ST depression in inferior leads	Not done	Not done	Mid LCX	RCA	PCI+aortic valve replacement	Unremarkable 5-year follow-up
6	Kawasaki disease	Prolonged PR interval			Not done	Not done	Proximal RCA		Aspirin and IV immunoglobulin for Kawasaki disease	Unremarkable 4-year follow-up
7	Chest pain		Normal LV wall motion, left ventricular EF: 60%	Stress ECG: ST depression in V1-V4+myocardial perfusion imaging: reversible ischemia in the anterior wall	Not done	Not done	Proximal RCA	LAD	PCI	Unremarkable 3-year follow-up on the woven artery. However, distal LAD developed 70% stenosis which required medical treatment
8	Atypical left arm pain	Old inferior infarction and T-wave inversion in V5-v6	Normal systolic function, inferior wall akinesis, and mild mitral regurge	Stress and rest Tc 99 m sestamibi scan: stress-induced myocardial ischemia in inferior and lateral leads	Not done	Not done	RCA	RCA	Medical treatment	Unremarkable 2-year follow-up
9	ACS	Normal	LV hypertrophy, mild diastolic dysfunction, and hypokinesia of the posterolateral wall with an ejection fraction of 55%		Not done	Not done	LAD, LCX, and OM2	LAD, LCX, and OM2	PCI	Unremarkable 1-year follow-up
10	ACS	ST depression in lead II, III, aVF, and V4-V6	LV hypokinesis, mild mitral insufficiency		Not done	Not done	Distal RCA	LM, LAD, LCX, and RCA	Medical treatment+CABG operation	Unremarkable postop
11	Chest pain	Normal	Normal wall motion, left ventricular EF: 65%	Stress ECG: ST-segment depression+thallium-201 myocardial perfusion imaging: no ischemia	Not done	Not done	LAD, LCX, RCA		Medical treatment+smoking cessation	
12	Chest pain	Sinus tachycardia, RBBB with marked ST-segment depression in precordial leads	Global hypokinesis, left ventricular EF: 30%		Not done	Not done	Proximal-distal RCA, proximal-mid LAD			
13	Incidental	Abnormal Q waves in leads V1–V6	Severe hypokinesia in the apical and anterior walls of the left ventricle, left ventricular EF: 45%	Infarction in the anterior and apical walls of the left ventricle, without any myocardial ischemia	Done	Not done	Proximal-mid LAD, proximal DI branch	LAD	Medical treatment	
14	ACS	Normal	Normal LV wall motion, left ventricular EF: 60%	Dobutamine stress echo: reversible ischemia in inferior wall	Not done	Not done	RCA	RCA due to stenosis before the woven segment	Medical treatment	
15	Chest pain			SPECT: stress-induced ischemia in the inferior wall of LV	Done	Not done	Mid-RCA	Distal RCA stenosis, CTO of PDA	CABG	
16	ACS				Not done	Not done	Proximal LAD, RCA, OM, and 1st diagonal	LAD	CABG	Unremarkable postop
17	Chest pain	Negative T waves in leads DII, DIII, and aVF	Normal LV wall motion, left ventricular EF: 65%	Myocardial perfusion imaging: no ischemia in the anterior wall	Not done	Not done	Distal LAD	LCX	Medical treatment	Unremarkable 1-year follow-up
18	Chest pain	Q waves and extrasystoles on D3 and aVF	Akinesia at inferior and posterior walls, left ventricular EF: 44%	Inferior wall ischemia was detected which approximately refers to 14% of the left ventricle	Not done	Not done	Mid-RCA	RCA	PCI	
19	Chest pain	Incomplete LBBB	Normal systolic function, LV hypertrophy	Myocardial perfusion imaging: reversible ischemia in inferior and posterior walls	Not done	Not done	RCA		Medical treatment	
20	Palpitations	Atrial flutter	Akinesia in the anterior and apical walls, severe hypokinesia in the other LV walls, and left ventricular EF: 22.5%	Stress-induced ischemia and a fixed low uptake in the anterior and apical walls	Not done	Done	Proximal-mid LAD		Cavotricuspid isthmus (CTI) ablation	
21	Sudden cardiac death	Asystole which could not be converted back to sinus rhythm					Mid RCA	RCA		Death
22	Exertional shortness of breath				Not done	Not done	LCX	LCX	CABG+mitral valve repair	Unremarkable 4-week follow-up
23	Incidental [symptoms of acute ischemic stroke of embolic origin]	Q waves in inferior leads	Akinesia in inferior and inferobasal walls, mild mitral regurgitation, and left ventricular EF: 40%	Myocardial perfusion scintigraphy: inferior wall fixed hypoperfusion, infarct, and mild peri-infarct ischemia	Not done	Not done	RCA	RCA	Medical treatment	Unremarkable 2-year follow-up
24	Congestive heart failure	Normal	Normal left ventricular EF		Done	Not done	LAD		Medical treatment	
	ACS		Regional inferior wall motion abnormality		Done	Not done	Distal RCA	RCA	PCI	
	Chest pain	Inverted T waves on precordial and inferior leads	NA		Done	Not done	Proximal RCA	LAD	PCI	Unremarkable 1-year follow-up
25	ACS	ST-segment elevation in the inferior leads	Normal LV wall motion, left ventricular EF: 59%		Not done	Not done	Proximal-mid RCA	RCA	CABG	
26	Chest pain	Atrial fibrillation in rhythm and Q wave in the inferior leads	Left ventricular EF: 40%		Not done	Done	RCA	RCA	PCI	Unremarkable 9-month follow-up. LVEF improved by 4% (44%)
27	Chest pain				Done	Done	RCA			
28	Exertional shortness of breath		Rheumatic heart disease		Not done	Not done	Middistal RCA	LAD	CABG+mitral valve replacement	
29	ACS	ST-segment elevation in the anterior V1-V5 leads	Akinesia at anterior walls, left ventricular EF: 54%		Done	Not done	LAD	LAD	PCI	Unremarkable 4-year follow-up
30	ACS			Ischemia in the inferior wall	Done	Not done	RCA	RCA	PCI	
31	Chest pain				Not done	Not done	LAD, LCX, RCA	LAD	PCI	Unremarkable 3-year follow-up
32	ACS [referred]				Done	Not done	RCA	RCA	PCI	
33	Incidental	Normal	Normal LV systolic function, left ventricular EF: >55%, moderately severe pulmonary hypertension, and mild dilatation of the right atrium	Not performed	Done	Done	RCA	RCA	PCI	Unremarkable 3-month follow-up

## Abbreviations

WCAA: Woven coronary artery anomaly
RHD: Rheumatic heart disease
PCI: Percutaneous intervention
PTCA: Percutaneous coronary intervention
CABG: Coronary artery bypass graft
OCT: Optical coherence tomography
FFR: Functional flow reserve
IVUS: Intravenous ultrasound
TIMI: Thrombolysis in Myocardial Infarction
RCA: Right coronary artery
LAD: Left anterior descending
LCX: Left circumflex
IHD: Ischemic heart disease.
